# Ion-pair compounds of diacerein for enhancing skin permeability *in vitro*: the compatibility–permeability relationship of counter ion and diacerein

**DOI:** 10.1080/10717544.2022.2032877

**Published:** 2022-02-11

**Authors:** Yan Liang, Manzhen Duan, Wei Yi, Teng Zhang, Yonggang Wang, Zhiming Wu, Huaibo Tang

**Affiliations:** a Department of Pharmacy, School of Chemistry, Xiangtan University, Xiangtan, China; b Department of Pharmaceutical Engineering, School of Chemical Engineering, Xiangtan University, Xiangtan, China

**Keywords:** Diacerein, ion-pair, transdermal, molecular simulation

## Abstract

This research aimed to investigate how the relationship between counter ion and diacerein (DCN) exerts an effect on the skin penetration of DCN ion-pair compounds. After the ion-pair compounds were formed by DCN and organic amines with different functional groups, the hydrogen bond of these compounds was confirmed by Fourier-transform infrared (FTIR) spectroscopy and molecular docking. The skin of porcine ears was employed to conduct the *in vitro* skin penetration, DCN – triethanolamine was the most potential candidate with the *Q*_24h_ of 7.89 ± 0.38 µg/cm^2^ among organic amines with different functional groups. Whereas among the homologous fatty amine, the most permeable compound was DCN – lauryl amine with the *Q*_24h_ of 11.28 ± 0.48 µg/cm^2^. Molecular simulation was employed to explore the relationship between counter ion and DCN. It was revealed by the bind energy curve that DCN had the strongest compatibility with triethanolamine among organic amines and laurylamine (N_12_) among fatty amines. It was amazingly found that the *in vitro* permeation fluxes of DCN ion-pair compounds would increase with enhancing the compatibility of counter ion and DCN. These findings broadened our understanding of how the relationship between drug and counter ion affects the skin penetration of ion-pair compounds.

## 1. Introduction

**Figure 1.**
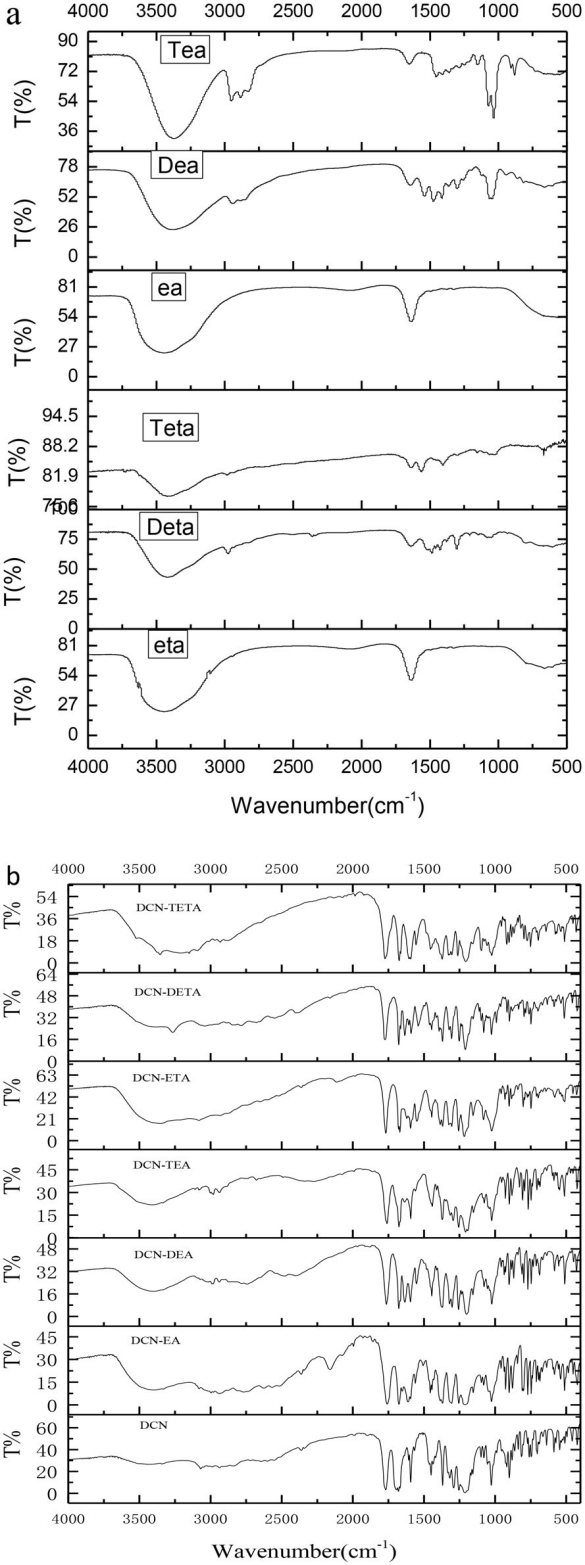
The FTIR spectra of (a) organic amine; (b) DCN and its ion-pair compounds.

Diacerein (DCN, Figure 1), a prodrug of rhein, is one of the few drugs that can effectively defer the inflammatory progression of osteoarthritis in the market (Kitadai et al. 2006; Dhaneshwar et al. 2013). It can selectively inhibit the activity of interleukin-1β (IL-1β) (Mahajan et al., 2006), reduce the expression of matrix metalloproteinases (Domagala et al., 2006) and repair cartilages by stimulating the expression of transforming growth factor β_1_ (TGF-β_1_) and β_2_ (TGF-β_2_) in arthral chondrocytes (Felisaz et al., 1999). Unfortunately, oral administration of DCN has low bioavailability (Walke et al., 2011; Rehman et al., 2015) and gastrointestinal adverse effects (Spencer & Wilde, 1997).

Drugs topically administrated on the skin of joints could be effectively distributed to the subjacent articular cavity of the applied site, which had excellent curative effects on osteoarthritis (Higaki et al., 2005; Shinkai et al., 2008; Tang et al., 2014; Wu et al., 2017). However, the skin is an effective barrier that prevents drugs from entering the body. It is generally accepted that the skin is mainly made up of the lipophilic stratum corneum (SC) and the aqueous viable skin including epidermis and dermis (Delgado-Charro & Guy, 2014). Therefore, it is helpful for drugs having the appropriate solubility to overcome the barrier of skin. Unfortunately, DCN has poor solubility both in water and non-polar solvent. In order to enhance the flux of skin penetration, niosomes (El-Say et al., 2016), bilosomes (Aziz et al., 2018a), elastosomes (Aziz et al., 2018b), and nanoemulsion (Chattopadhyay & Datta, 2018) had been developed for DCN in recent years. But these pharmaceutical methods had some shortcomings, such as instability and complicated preparation process (Table 1).

**Table 1.** The chemical structure of DCN and counter ion.

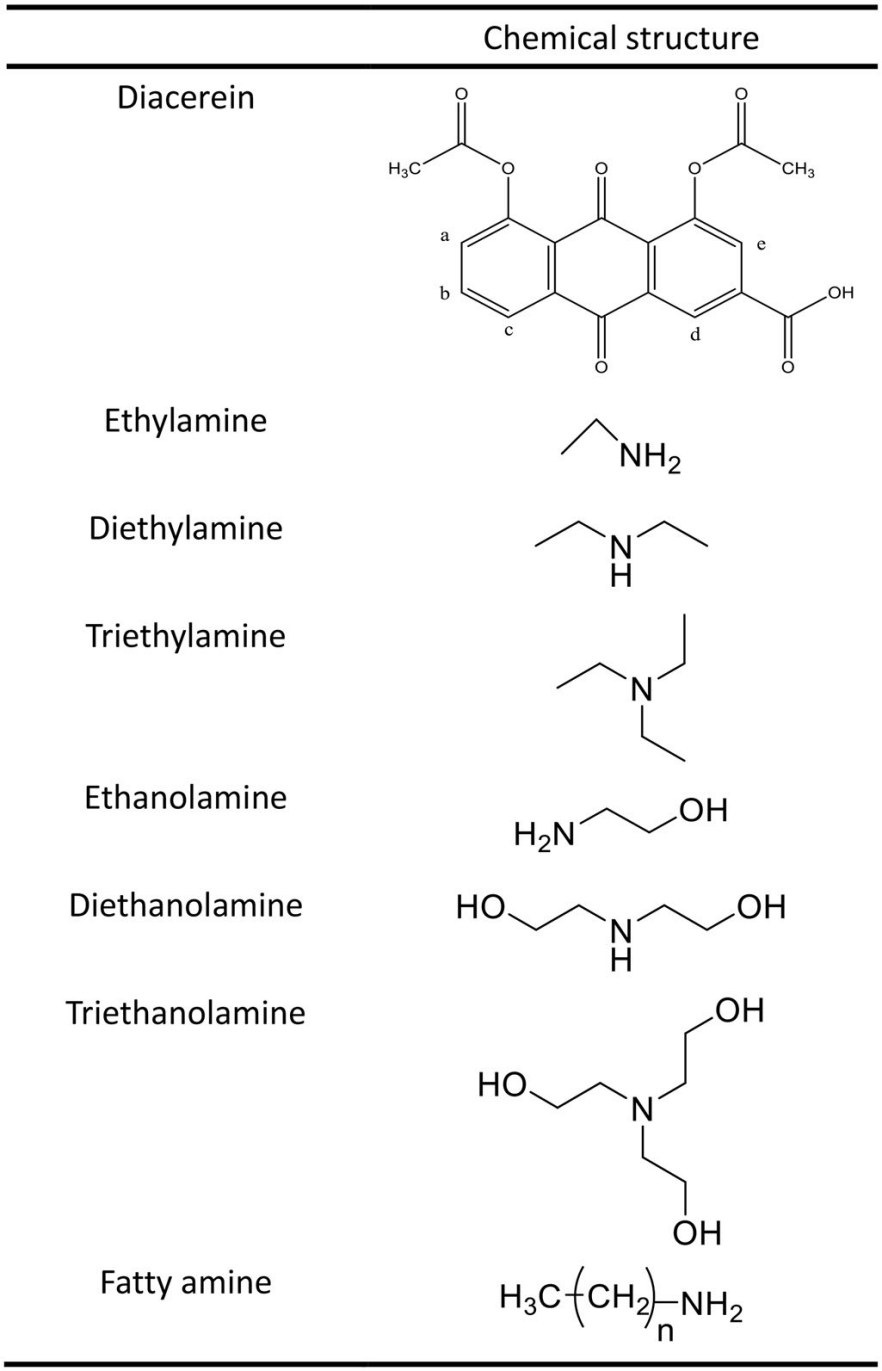

Without change of drug pharmacologic actions, ion-pair strategy is usually employed to enhance the skin penetration, which can form the hydrogen bond between drug and counter ions (Ogiso & Shintani 1990; Xi et al., 2012a). The skin permeability of the ion-pair compound would be obviously influenced by some factors, such as physicochemical properties (Song et al., 2012, 2016), stability (Xi et al., 2012b), ionizability in viable epidermis (Zhao et al., 2017), and polar surface area (Cui et al., 2015). Although the influence of ion-pair compounds on skin penetration had been summarized in previous reports, it still deserved to investigate the influence of the relationship between drug and counter ion on the skin penetration of ion-pair compounds.

This research aimed to how the relationship between counter ion and DCN exerts an effect on the skin penetration of DCN ion-pair compounds. First, the organic amines with different functional groups were selected to form ion-pair compounds with DCN, and the hydrogen bond of these compounds was confirmed by FTIR and molecular simulation. Subsequently, the *in vitro* skin permeation experiments of these compounds were investigated. In order to avoid different functional groups of ion-pair compounds influencing on skin permeability, a series of homologous fatty amines were also selected to form ion-pair compounds with DCN, and then the *in vitro* permeation experiments of these compounds with the same functional groups were also conducted. Finally, the relationship of DCN and counter ion was further explored by blends module in the Material Studio 8.0 software (Accelrys, San Diego, CA).

## 2. Materials and methods

### 2.1. Materials

Diacerein was obtained from Xi'an Tianfeng Biotechnology Co., Ltd. (Xi'an, China). Aminoethane (ea), diethyl amine (Dea), triethyl amine (Tea), aminoethyl alcohol (eta), diethanol amine (Deta), triethanol amine (Teta), and isopropyl myristate (IPM) were purchased from Chemical Reagent Co., Ltd. (Shanghai, China). Aminohexane (N_6_), n-octyl amine (N_8_), decyl amine (N_10_), lauryl amine (N_12_), tetradecyl amine (N_14_), cetyl amine (N_16_), and n-octadecyl amine (N_18_) were supplied by Shanghai McLaren Biochemical Technology Co., Ltd. (Shanghai, China). HPLC grade acetonitrile was provided by Tianjin Comio Chemical Reagent Co., Ltd. (Tianjin, China). All other chemicals were of analytical grade.

### 2.2. Preparation of DCN ion-pair complexes

DCN and equimolar of organic amines were dissolved in acetone, and then mixed by magnetic stirrer bars for 24 h at room temperature. Subsequently, the mixture was filtered and washed with anhydrous ethanol to remove excess organic amines. Finally, the obtained DCN ion-pair compounds were dried by vacuum drying.

### 2.3. FTIR spectroscopy studies

An appropriate amount of DCN and its ion-pair complexes were mixed with KBr. After that the mixtures were ground, dried and then pressed as tablets. An appropriate amount of amine was directly painted on the infrared window slice of potassium bromide (Tianjin Tianpu Instrument, Tianjin, China). The FTIR spectra was recorded on a Nicolet 6700 spectrometer (Thermo Fisher Scientific, Waltham, MA) with the scanning range of 4000–400 cm^−1^ and a resolution of 4 cm^−1^.

### 2.4. DSC experiments

SDT Q600 instrument (TA Instruments, New Castle, DE) was used to characterize the melting point of DCN and its ion-pair complexes. About 4 mg of drugs were placed in a standard aluminum crucible with a heating range of 25–350 °C and a heating rate of 10 °C·min^−1^. All samples were tested under the protection of 50 mL·min^−1^ of nitrogen flow.

### 2.5. Solubility experiments

The excess DCN and its ion-pair complexes were separately added to 2.0 mL solvent in a sealed polypropylene micro-vials. The obtained complexes were vibrated by ultrasonic for 10 min then continuously shaken in a water bath at 32 °C until equilibrium. At predetermined time interval 48 h, 0.3 mL sample was withdrawn, the samples were determined by HPLC after being filtered through the membrane of 0.45 μm and diluted if necessary.

### 2.6. Apparent distribution coefficient studies

The classic shake-flask method was used to determine the apparent distribution coefficient (Log *P*). DCN and its ion-pair complexes were added into n-octanol, then mixed with equal volume distilled water. The samples were shaken for 48 h at 32 °C and centrifuged at 10,000 rpm for 10 min. After phase separation, the concentrations of each drug in both water and n-octanol layer were analyzed by HPLC.

### 2.7. *In vitro* permeation experiments

According to the description in our previous report (Xu et al., 2011), the porcine ears immediately obtained from the killed pig were supplied by local slaughterhouse (Xiangtan, China). The hair of porcine ears was carefully cut off with animal hair clippers. Then, skin grafting knife (Medical Equipment Factory of Shanghai Medical Instruments Co., Ltd., Shanghai, China) was employed to separate the skin and subcutaneous fat, and the skin of thickness in range of 0.6 ± 0.1 mm was obtained. The skin was kept frozen at −20 °C no more than 2 weeks.

Two-chamber Franz diffusion cells (receiver volume of 6.5 mL, effective diffusion area of 2.8 cm^2^) were used for the *in vitro* transdermal experiments. The prepared skin was placed between the cell halves with the SC facing the donor cell. The receptor compartment was filled with phosphate buffered saline (pH 7.4), which was put in a water bath at 32 ± 0.5 °C and stirred with a magnetic bar at 300 rpm. The saturated solution of DCN and its ion-pair compounds in isopropyl myristate was filled in the donor cell, and a glass sheet was employed to cover the supply chamber during the *in vitro* permeation experiments. Since the experiment started, 1.0 mL sample was withdrawn from the receptor compartment at predetermined time interval 2, 4, 6, 8, 10, 12, 14, and 24 h. Subsequently, the equal volume of fresh PBS was replenished. These investigations were performed with different individual skin at least in triplicate.

### 2.8. HPLC analysis

The samples were determined by an LC-20A HPLC system (SHIMADZU, Kyoto, Japan). The separation was achieved on an InertSustain C18 column (4.6 mm × 250 mm, 5 µm, SHIMADZU, Kyoto, Japan) with the flow rate keeping at 1.0 mL/min, the column temperature maintaining at 40 °C and mobile phase consisting of methanol:water (25:75, v/v) with 0.4% phosphoric acid in water. Injection volume for each run was 50 μL, and detection wavelength was 254 nm.

### 2.9. Molecular simulation

At first smart algorithm (cascade of steepest descent, quasi-Newton methods, and conjugate gradient) was employed to obtain the optimal geometry of Str ion-pair compound and the organic amine. Molecular docking was performed in blends module using Materials Studio Version 8.0 (Accelrys, San Diego, CA), and COMPASS force field was conducted throughout the whole simulation processes (Cui et al., 2015; Zhao et al., 2017).

Binding energy curves of two components in blends simulation is an effective tool to distinguish their compatibility (Wang et al., 2016). The role property of the components can be distinguished in evaluating binding energies: one component acts as a base role (*E*_bb_) and the other serve as a screen role (*E*_ss_). In order to investigate the compatibility of Str and its ion-pair compounds with skin, molecular simulation was carried out in blends module of Materials Studio Version 7.0 (Accelrys Inc., San Diego, CA), and COMPASS force field was performed throughout the whole simulation processes.

## 3. Results

### 3.1. FTIR spectra

**Figure 2.**
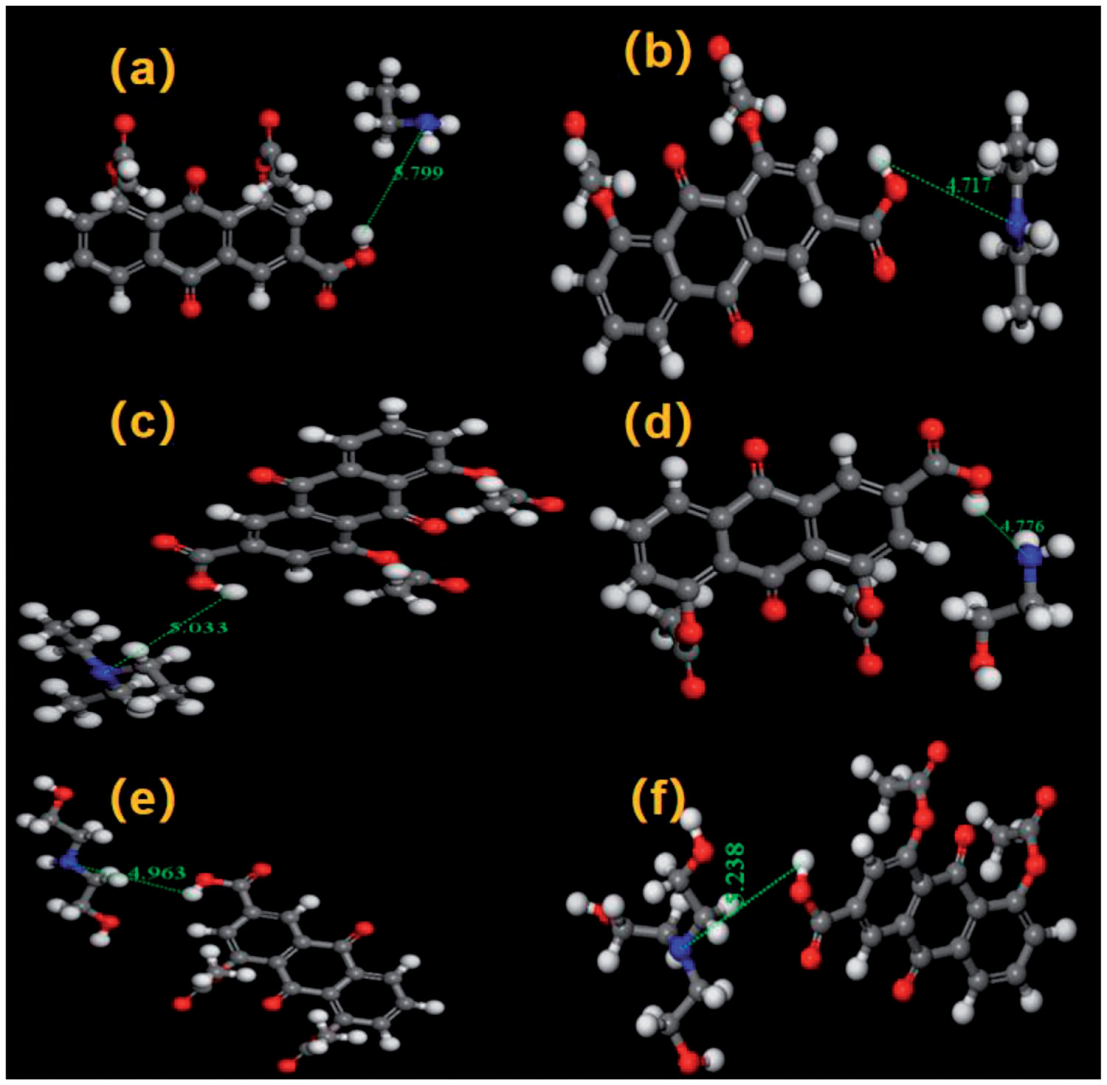
Minimum energy of DCN ion-pair compounds forming with A (a) EA, (b) DEA, (c) TEA, (d) ETA, (e) DETA, and (f) TETA.

FTIR spectroscopy could offer the evidence of the proton transfer from the carboxylic acid of DCN to the amine. The FTIR spectra of DCN and its ion-pair complexes are shown in Figure 2. The stretching vibration of the N–H group was among 3500–3200 cm^−1^ (Silverstein et al., 2014). After DCN formed ion-pair compounds with organic amine, a new continuous broad absorption peak appeared among 3300–2000 cm^−1^. DCN has the strong narrow peak at 1693.44 cm^−1^, which belongs to the C=O of the carboxyl group (Arunan et al., 2011). But this strong narrow peak of DCN ion-pair compounds had red shift.

### 3.2. The physicochemical properties of DCN ion-pair compounds with organic amines

The physicochemical properties of DCN ion-pair compounds including melt point, solubility and Log *P* value are presented in Table 2. After the DCN ion-pair compounds were formed by DCN and six organic amines, the melt point decreased obviously. The Log *P* value of the DCN was 1.50, while the Log *P* value of those DCN ion-pair compounds decreased to −0.4 to –0.1. The solubility of DCN in water was no more than 10 µg/mL; however, the solubility of DCN ion-pair compound in water increased to more than 3000 µg/mL. These indicated that the polarity of DCN ion-pair compounds increased significantly. Isopropyl palmitate is a lipophilic solvent with low dielectric constant (*ε*_r_=3.18), which is frequently used as a vehicle solution in transdermal research of ion-pair compounds (Xi et al., 2012a,b; Cui et al., 2015). Thus, isopropyl myristate was selected as the donor solution in this experiment.

**Table 2.** Physicochemical properties of DCN and DCN ion-pair compounds.

	Melt point (°C)	Log *P*	Equilibrium solubility (μg/mL)		
			IPM	PBS (pH 7.4)	Water
DCN	260.84	1.50 ± 0.01	83.59 ± 18.05	1441.87 ± 5.58	8.29 ± 0.27
DCN-ea	243.05	–0.39 ± 0.007	92.80 ± 14.10	12155.69 ± 81.21	6928.52 ± 4.80
DCN-Dea	176.57	–0.32 ± 0.007	410.37 ± 20.56	18473.86 ± 91.09	5453.36 ± 27.84
DCN-Tea	172.47	–0.27 ± 0.006	1385.02 ± 13.68	8187.55 ± 44.36	3232.52 ± 12.61
DCN-eta	148.83	–0.25 ± 0.005	121.56 ± 1.20	6099.91 ± 24.71	3059.63 ± 35.77
DCN-Deta	149.10	–0.22 ± 0.006	115.89 ± 16.60	11105.50 ± 18.51	3998.30 ± 12.50
DCN-Teta	153.92	–0.13 ± 0.005	99.38 ± 12.80	2514.24 ± 10.27	6374.94 ± 14.60

### 3.3. The skin permeation of DCN ion-pair compounds with organic amines

After the ion-pair compounds were formed by DCN and organic amines with different groups, the *in vitro* penetration experiments of DCN and these compounds were conducted. As shown in Table 3, the transdermal permeation rate of DCN ion-pair compounds was significantly higher than that of DCN, and the *Q*_24h_ of DCN ion-pair compounds with alkanolamine was higher than those compounds with alkylamines. Among six DCN ion-pair compounds, DCN-Teta has the highest transdermal permeation rate. The different skin permeability of DCN ion-pair compounds with organic amines was perhaps caused by the hydrogen-bonding potential of the intercellular lipid and ion-pair compounds because of the counter ion with different types and numbers of organic amine (Cui et al., 2015).

**Table 3.** Skin permeation data of DCN and its ion-pair compounds with different groups.

Samples	*Q*_24h_^a^ (µg/cm^2^)	*J*_ss_^b^ (µg/cm^2^⋅h^–1^)	*T*_lag_^c^ (h)
DCN	1.83 ± 0.22	0.06 ± 0.01	2.68 ± 0.78
DCN-ea	2.17 ± 0.30	0.08 ± 0.02	1.79 ± 0.66
DCN-Dea	2.82 ± 0.49	0.10 ± 0.01	4.12 ± 1.14
DCN-Tea	2.85 ± 0.64	0.11 ± 0.04	3.39 ± 0.55
DCN-eta	3.04 ± 0.67	0.09 ± 0.04	1.41 ± 0.56
DCN-Deta	4.03 ± 0.71	0.11 ± 0.02	2.16 ± 0.55
DCN-Teta	7.89 ± 0.38	0.17 ± 0.05	1.52 ± 0.48

^a^ *Q*_24h_, the cumulative amount of drug permeated per unit area at 24 h.

^b^ *J*_ss_, the steady-state flux obtained from the slope of the linear portion of the plots.

^c^ *T*_lag_, the extrapolated value of the linear portion of the permeation curve to the abscissa.

### 3.4. The skin permeation of DCN ion-pair compounds with fatty amines

In order to avoid different functional groups introducing to DCN ion-pair compounds, a series of homologous fatty amines were selected to form ion-pair compounds with DCN. The results of the *in vitro* transdermal penetration for DCN and those results are presented in Table 4. As the number of carbon atoms increased, the transdermal permeation rate of DCN ion-pair compounds would increase slowly, then decrease gradually, and attain the peak value at 12 carbon atoms. It was also found that the *Q*_24h_ of DCN-N_12_ was higher than that of DCN-Teta.

**Table 4.** Skin permeation data of DCN and its ion-pair compounds with fatty amines.

Samples	*Q*_24h_^a^ (µg/cm^2^)	*J*_ss_^b^ (µg/cm^2^⋅h^–1^)	*T*_lag_^c^ (h)
DCN	1.83 ± 0.22	0.06 ± 0.01	2.68 ± 0.78
DCN-N_6_	3.86 ± 0.21	0.10 ± 0.02	1.52 ± 0.36
DCN-N_8_	4.30 ± 0.63	0.14 ± 0.03	2.15 ± 0.22
DCN-N_10_	6.41 ± 0.23	0.20 ± 0.03	1.38 ± 0.14
DCN-N_12_	11.28 ± 0.48	0.34 ± 0.02	2.50 ± 0.18
DCN-N_14_	7.03 ± 1.56	0.24 ± 0.05	1.31 ± 0.11
DCN-N_16_	6.44 ± 0.36	0.22 ± 0.01	1.28 ± 0.23
DCN-N_18_	5.97 ± 1.25	0.20 ± 0.01	1.42 ± 0.52

^a^ *Q*_24h_, the cumulative amount of drug permeated per unit area at 24 h.

^b^ *J*_ss_, the steady-state flux obtained from the slope of the linear portion of the plots.

^c^ *T*_lag_, the extrapolated value of the linear portion of the permeation curve to the abscissa.

### 3.5. Molecular simulation

**Figure 3.**
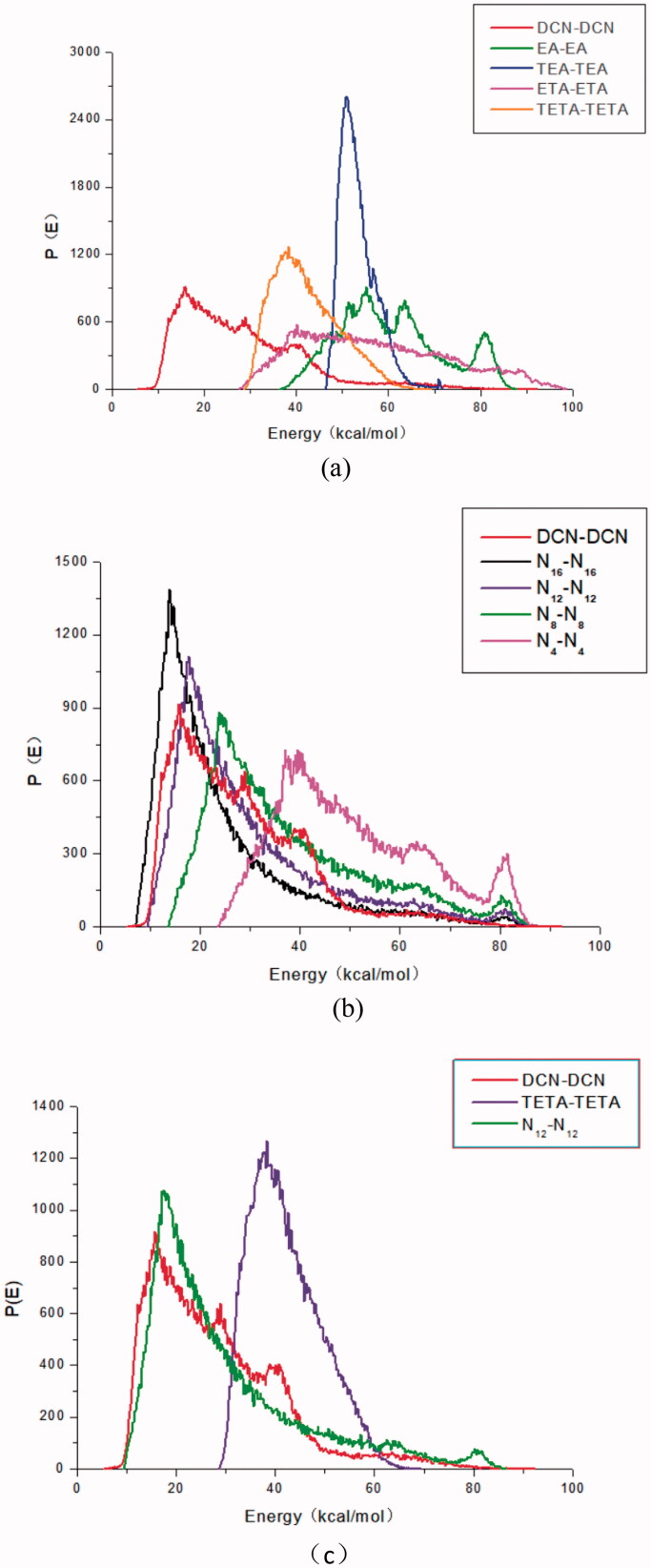
The binding energy distributions curves of DCN and (a) organic amines with different functional groups; (b) fatty amines; (c) triethanolamine and lauryl amine.

The optimal geometry of DCN and counter ions was obtained by smart algorithm. Then molecular docking was employed to confirm the hydrogen bond of DCN ion-pair compounds. As shown in Figure 3, the hydrogen bond of DCN ion-pair compounds was formed between DCN and all counter ions. In detail, the formation of the hydrogen bond was the proton transfer from the deprotonated acids anion (RCOO^−^) of DCN to the protonated cation (^+^HNR) of counter ions.

Usually, the compatibility of two components can be judged by their binding energy curves: one component acts as a base role (*E*_bb_) and the other component serves as a screen role (*E*_ss_). In the progress of molecular simulation, DCN and counter ions are set to a base role (*E*_bb_, red line) and a screen role (*E*_ss_, other colors line), respectively. The binding energy curves of the two substances were relatively similar, the compatibility of the two substances was better (Wang et al., 2016). Among organic amines with different functional groups, the binding energy curve of triethanolamine was most similar to that of DCN, as shown in Figure 3(a). Thus it could be concluded that triethanolamine had the best compatibility with DCN. As shown in Figure 3(b), it could also be concluded that laurylamine (N_12_) had the best compatibility with DCN among selected fatty amine. As shown in Figure 3(c), it was also found that binding energy distribution curves of laurylamine and DCN was more similar, which indicated that laurylamine (N_12_) had stronger compatibility with DCN than triethanolamine.

## 4. Discussion

Ion-pair compounds have a special type of hydrogen bond with a proton transferring between drug and counter ion (Xi et al., 2012a,b). Organic amines could offer a pair lone electron, and the proton would be provided by the carboxyl group of DCN. The DCN ion-pair compound of the C=O on carboxyl group had a red shift. For that reason, it was concluded that the hydrogen bond was formed at the carboxyl position of DCN. A continuous broad absorption peak appeared among 3300–2000 cm^−1^, which was attributed to the tunneling effect of intermolecular hydrogen bonds during the transfer of protons. The hydrogen bond of DCN ion-pair compounds at the carboxyl position of DCN was further confirmed by molecular docking.

The Log *P* value of ion-pair compounds is a pivotal factor that influences the skin permeation fluxes (Song et al., 2016; Zhao et al., 2017). However, the Log *P* could not always reflect the results of permeation fluxes. After DCN formed the ion-pair compounds with organic amines possessing different functional groups, the Log *P* value of these compounds decreased obviously. The correlation between skin permeability fluxes of DCN compounds and Log *P* was consistent with some literature reports (Song et al., 2012; Xi et al., 2012b). However, as for some drugs with appropriate Log *P* value, the skin permeability fluxes of the ion-pair compounds had negative relationship with their Log *P* value (Song et al., 2012, 2016).

The penetrant diffusion coefficient of SC would be obviously affected by the hydrogen bond of the intercellular lipids and penetrant (Hadgraft, 2001). The intercellular lipids is the main barrier of skin, which have many donors and receptors of hydrogen bond (Hadgraft, 2001; Gary et al., 2012). It was reported that the counter ions with lower hydrogen-bonding potential tended to achieve the better effect of skin permeability (Cui et al., 2015). The skin permeability of DCN and organic amine ion-pair compounds maybe affected by the different hydrogen bond of the intercellular lipids and the ion-pair compounds due to the counter ion with different types and numbers of organic amine. As for the ion-pair compounds of DCN and fatty amines, their skin permeability should not be influenced by the different hydrogen bond of the intercellular lipids and their ion-pair compounds. However, the transdermal permeation rate of these compounds increased slowly, then decreased gradually with the carbon atoms of counter ion increasing.

The properties of ion pairs are affected not only by the properties of drugs, but also by the properties of counter ions. It is worth exploring how the relationship between counter ion and DCN effect on the skin penetration of DCN ion-pair compounds. It was found by the bind energy curve that DCN had the strongest compatibility with triethanolamine among organic amines with different functional groups and laurylamine (N_12_) among fatty amines. Laurylamine had stronger compatibility of with DCN than triethanolamine. Among organic amines with different functional groups, DCN-Teta was the most potential candidate. The transdermal permeation rate of DCN-fatty acid compounds would increase slowly with the carbon atoms of counter ion increasing, attain the peak at 12, then decreased gradually. The skin permeation rate of DCN-N_12_ was higher than that of DCN-Teta. It was amazingly found that the *in vitro* permeation fluxes of DCN ion-pair compounds would increase with enhancing the compatibility of counter ion and DCN.

Ion-pair compounds pass through SC more easily than individual ions because the central ion and an oppositely charged ion behave as a single-unit behavior via the electrostatic interactions (McNaught & Wilkinson, 1997; Cavallari et al., 1998; Fini et al., 1999; Cristofoli et al., 2021). However, these electrostatic interactions of the hydrogen bond are very weak, the hydrogen bond of ion-pair compounds breaks and forms on an extremely short timescale (Song et al., 2016). It was reported that the longer the ion-pair lifetime, the higher the skin permeation flux (Xi et al., 2012b). Perhaps the life time of DCN ion-pair compounds would be extended because the electrostatic interaction of counter ion and DCN would increase with their compatibility enhancing (Song et al., 2016; Cristofoli et al., 2021).

## 5. Conclusions

The hydrogen bond of DCN ion-pair compounds formed at the carboxyl position of DCN, and the skin permeation fluxes of DCN ion-pair compounds would increase with the enhancement of the compatibility of counter ions and DCN. These findings broadened our knowledge of how the relationship of drug and counter ion affects percutaneous penetration of ion-pair compounds.

## References

[CIT0001] Arunan E, Desiraju GR, Klein RA, et al. (2011). Defining the hydrogen bond: an account (IUPAC technical report). Pure Appl Chem 83:1619–36.

[CIT0002] Aziz D, Abdelbary A, Elassasy AI. (2018a). Investigating superiority of novel bilosomes over niosomes in the transdermal delivery of diacerein: *in vitro* characterization, ex vivo permeation and in vivo skin deposition study. J Liposome Res 29:73–85. 29355060 10.1080/08982104.2018.1430831

[CIT0003] Aziz D, Abdelbary A, Elassasy A. (2018b). Fabrication of novel elastosomes for boosting the transdermal delivery of diacerein: statistical optimization, ex-vivo permeation, in-vivo skin deposition and pharmacokinetic assessment compared to oral formulation. Drug Deliv 25:815–26. 29557244 10.1080/10717544.2018.1451572 PMC6058680

[CIT0004] Cavallari C, Passerini N, Fini A, et al. (1998). Partition of diclofenac salts as ion-pairs. Eur J Pharm Sci 6:S63.

[CIT0005] Chattopadhyay H, Datta S. (2018). Transdermal delivery of diacerein with homing carrier glucosamine sulphate laden in oil-in-water nanoemulsion. Mater Today Proc 5:9690–7.

[CIT0007] Cristofoli M, Kung CP, Hadgraft J, et al. (2021). Ion pairs for transdermal and dermal drug delivery: a review. Pharmaceutics 13:909. 34202939 10.3390/pharmaceutics13060909 PMC8234378

[CIT0008] Cui H, Quan P, Zhao H, et al. (2015). Mechanism of ion-pair strategy in modulating skin permeability of zaltoprofen: insight from molecular-level resolution based on molecular modeling and confocal laser scanning microscopy. J Pharm Sci 104:3395–403. 26349639 10.1002/jps.24543

[CIT0009] Delgado-Charro MB, Guy RH. (2014). Effective use of transdermal drug delivery in children. Adv Drug Deliv Rev 73:63–82. 24333231 10.1016/j.addr.2013.11.014

[CIT0010] Dhaneshwar S, Patel V, Patil D, et al. (2013). Studies on synthesis, stability, release and pharmacodynamic profile of a novel diacerein-thymol prodrug. Bioorg Med Chem Lett 23:55–61. 23218603 10.1016/j.bmcl.2012.11.016

[CIT0011] Domagala F, Martin G, Bogdanowicz P, et al. (2006) Inhibition of interleukin-1beta-induced activation of MEK/ERK pathway and DNA binding of NF-kappaB and AP-1: potential mechanism for diacerein effects in osteoarthritis. Biorheology 43:577–87. 16912429

[CIT0012] El-Say KM, Abd-Allah FI, Lila AE, et al. (2016) Diacerein niosomal gel for topical delivery: development, *in vitro* and *in vivo* assessment. J Liposome Res 26:57–68. 25853339 10.3109/08982104.2015.1029495

[CIT0013] Felisaz N, Boumediene K, Ghayor C, et al. (1999). Stimulating effect of diacerein on TGF-beta_1_ and beta_2_ expression in articular chondrocytes cultured with and without interleukin-1. Osteoarthritis Cartilage 7:255–64. 10329300 10.1053/joca.1998.0199

[CIT0014] Fini A, Fazio G, Gonzalez-Rodriguez M, et al. (1999). Formation of ion-pairs in aqueous solutions of diclofenac salts. Int J Pharm 187:163–73. 10502622 10.1016/s0378-5173(99)00180-5

[CIT0015] Gary PM, Simon CW, Sun Y. (2012). Mathematical modelling of percutaneous absorption. Curr Opin Colloid Interface Sci 17:72–166.

[CIT0016] Hadgraft J. (2001). Skin, the final frontier. Int J Pharm 224:1–18. 11512545 10.1016/s0378-5173(01)00731-1

[CIT0017] Higaki K, Nakayama K, Suyama T, et al. (2005). Enhancement of topical delivery of drugs via direct penetration by reducing blood flow rate in skin. Int J Pharm 288:227–33. 15620862 10.1016/j.ijpharm.2004.09.025

[CIT0018] Kitadai HK, Takahashi HK, Straus AH, et al. (2006). Effect of oral diacerein (DAR) in an experimental hip chondrolysis model. J Orthop Res 24:1240–8. 16705708 10.1002/jor.20180

[CIT0019] Mahajan A, Singh K, Tandon VR, et al. (2006). Diacerein: a new symptomatic slow acting drug for osteoarthritis. J Med Educ Res 8:173–5.

[CIT0020] McNaught AD, Wilkinson A. (1997). Compendium of chemical terminology. 2nd ed. Oxford (UK): Blackwell Scientific Publications.

[CIT0021] Ogiso T, Shintani M. (1990). Mechanism for the enhancement effect of fatty acids on the percutaneous absorption of propranolol. J Pharm Sci 79:1065–71. 2079652 10.1002/jps.2600791206

[CIT0023] Rehman M, Madni A, Ihsan A, et al. (2015). Solid and liquid lipid-based binary solid lipid nanoparticles of diacerein: *in vitro* evaluation of sustained release, simultaneous loading of gold nanoparticles, and potential thermoresponsive behavior. Int J Nanomedicine 10:2805–14. 25897224 10.2147/IJN.S67147 PMC4396646

[CIT0024] Shinkai N, Korenaga K, Mizu H, et al. (2008). Intra-articular penetration of ketoprofen and analgesic effects after topical patch application in rats. J Control Release 131:107–12. 18680771 10.1016/j.jconrel.2008.07.012

[CIT0025] Silverstein RM, Webster FX, Kiemle DJ, et al. (2014). Spectrometric identification of organic compounds. 8th ed, New Jersey: Willey.

[CIT0026] Song T, Quan P, Xiang R, et al. (2016). Regulating the skin permeation rate of escitalopram by ion-pair formation with organic acids. AAPS PharmSciTech 17:1267–73. 26762339 10.1208/s12249-015-0474-y

[CIT0027] Song W, Cun D, Xi H, et al. (2012). The control of skin-permeating rate of bisoprolol by ion-pair strategy for long-acting transdermal patches. AAPS PharmSciTech 13:811–5. 22639239 10.1208/s12249-012-9808-1 PMC3429664

[CIT0028] Spencer CM, Wilde MI. (1997). Diacerein. Drugs 53:98. 9010651 10.2165/00003495-199753010-00007

[CIT0029] Tang HB, Yan M, Li HD, et al. (2014). Dynamic detection of non-protein-bound strychnine and brucine in rabbit muscle and synovial fluid after topical application of total Strychnos alkaloid patches. Drug Test Anal 6:357–62. 23776063 10.1002/dta.1493

[CIT0030] Walke PS, Khairna PS, Narkhede MR, et al. (2011). Solubility enhancement of diacerein by mannitol solid dispersions. Int J Pharm Sci 3:261–4.

[CIT0031] Wang Y, Ren J, Zhang C, et al. (2016). Compatibility studies between amphiphilic pH-sensitive polymer and hydrophobic drug using multiscale simulations. RSC Adv 6:101323–33.

[CIT0032] Wu P, Liang Q, Feng P, et al. (2017). Novel brucine gel transdermal delivery system designed for anti-inflammatory and analgesic activities. Int J Mol Sci 18:757. 10.3390/ijms18040757 PMC5412342 28368343

[CIT0033] Xi H, Cun D, Wang Z, et al. (2012a). Effect of the stability of hydrogen-bonded ion pairs with organic amines on transdermal penetration of teriflunomide. Int J Pharm 436:857–61. 22796174 10.1016/j.ijpharm.2012.07.004

[CIT0034] Xi H, Wang Z, Chen Y, et al. (2012b). The relationship between hydrogen-bonded ion-pair stability and transdermal penetration of lornoxicam with organic amines. Eur J Pharm Sci 47:325–30. 22579706 10.1016/j.ejps.2012.04.017

[CIT0035] Xu L, Yang ZY, Tang HB, et al. (2011). Effect of chemical enhancers on the permeation of strychnine across pig skin *in vitro*. Central South Pharm 9:761–3.

[CIT0036] Zhao H, Liu C, Quan P, et al. (2017). Mechanism study on ion-pair complexes controlling skin permeability: effect of ion-pair dissociation in the viable epidermis on transdermal permeation of bisoprolol. Int J Pharm 532:29–36. 28830782 10.1016/j.ijpharm.2017.08.080

